# Multimodal combination of GC × GC-HRTOFMS and SIFT-MS for asthma phenotyping using exhaled breath

**DOI:** 10.1038/s41598-020-73408-2

**Published:** 2020-09-30

**Authors:** Pierre-Hugues Stefanuto, Delphine Zanella, Joeri Vercammen, Monique Henket, Florence Schleich, Renaud Louis, Jean-François Focant

**Affiliations:** 1 Organic and Biological Analytical Chemistry Group, MOLSYS Research Unit, University of Liège, Allée du 6 Août B6c, 4000 Liège, Belgium; 2 Interscience, Avenue J.E. Lenoir, Louvain-la-Neuve, Belgium; 3 Engineering, Industrial Catalysis and Adsorption Technology (INCAT), Ghent University, Ghent, Belgium; 4 Pneumology and Allergology, GIGA Research Group, CHU of Liège, University of Liege, Liège, Belgium

**Keywords:** Metabolomics, Diagnostic markers, Predictive markers, Asthma, Chronic obstructive pulmonary disease, Diagnostic markers, Predictive markers

## Abstract

Chronic inflammatory lung diseases impact more than 300 million of people worldwide. Because they are not curable, these diseases have a high impact on both the quality of life of patients and the healthcare budget. The stability of patient condition relies mostly on constant treatment adaptation and lung function monitoring. However, due to the variety of inflammation phenotypes, almost one third of the patients receive an ineffective treatment. To improve phenotyping, we evaluated the complementarity of two techniques for exhaled breath analysis: full resolving comprehensive two-dimensional gas chromatography coupled to high-resolution time-of-flight mass spectrometry (GC × GC-HRTOFMS) and rapid screening selected ion flow tube MS (SIFT-MS). GC × GC-HRTOFMS has a high resolving power and offers a full overview of sample composition, providing deep insights on the ongoing biology. SIFT-MS is usually used for targeted analyses, allowing rapid classification of samples in defined groups. In this study, we used SIFT-MS in a possible untargeted full-scan mode, where it provides pattern-based classification capacity. We analyzed the exhaled breath of 50 asthmatic patients. Both techniques provided good classification accuracy (around 75%), similar to the efficiency of other clinical tools routinely used for asthma phenotyping. Moreover, our study provides useful information regarding the complementarity of the two techniques.

## Introduction

According to the world health organization, asthma and chronic obstructive pulmonary disease (COPD) represent the most common chronic respiratory diseases, with respectively 235 and 65 millions of people concerned worldwide (WHO asthma and COPD Fact Sheets). These diseases are not curable. Various forms of treatment that help to dilate major airways and limit the shortness of breath can help control symptoms and increase the quality of life for patients. However, despite similar symptoms, different inflammation phenotypes exist for both diseases. As each phenotype requires different treatment approaches but are currently poorly diagnosed, about 20–30% of asthmatic patients are on the wrong medication. Better phenotyping is therefore needed to improve the efficacy of prescribed treatments and preserve patients from suffering unnecessary side effects^1^. It would allow quick adjustment of the treatment when needed. Moreover, it would provide insights regarding the impact of known and unknown risk factors, such as tobacco smoke, air pollution, occupational chemicals and dusts, and frequent lower respiratory infections during childhood^2^.

Currently, the phenotyping of asthma and COPD relies on invasive sputum analysis or empirical approaches^3^. Indeed, inflammatory phenotypes are defined by the American Thoracic Society based on the percentage of inflammatory cells present in the sputum^4^. However, sputum analysis is invasive, expensive, and not available in most of the medical center. Due to the difficulties linked to sputum analysis, this method is not suitable for frequent longitudinal monitoring of disease evolution^4^. Other diagnosis alternatives have been developed using the measurement of the fraction of exhaled nitric oxide (FeNO) or blood cell count^5,6^. Nevertheless, those techniques can only rule in eosinophilic phenotype, without differentiating between the others^4–6^. Recently, we validated a set of volatile markers present in the breath of asthmatic patients to differentiate between inflammatory phenotypes^7^. This discovery represents an important step forward in the quest of a non-invasive rapid diagnostic method.

The recent evolution of analytical technologies dedicated to separation and measurement of volatile organic compounds (VOCs) has reinforced the branch of “omics” sciences that focus on low molecular weight analytes. On one side, separation science based on multidimensional methods such as comprehensive two-dimensional gas chromatography (GC × GC) appeared as one of the methods of choice for the characterization of complex mixtures of (semi)volatile chemicals, especially when coupled to high-resolution time-of-flight mass spectrometry (HRTOFMS)^8^. On another side, direct introduction instruments such as selected ion flow tube MS (SIFT-MS) offers now the capacity to perform both targeted and non-targeted analyses within a few minutes (^9–11^). At the price of high cost equipment and limited adaptability to routine medical usage, GC × GC-HRTOFMS offers the possibility to almost completely characterize the VOC sample composition. This is of prime importance for chemical support of systems biology, but it also offers the possibility for the creation of specific lists of biomarkers of disease. For large scale screening, SIFT-MS can generate compositional patterns from direct sample introduction concurrently to other established routine medical actions. These two orthogonal approaches can therefore be considered for pathology screening in a population of patients and should ideally conduct to identical sample classifications. They however have never been directly used and compared over an identical set of patients.

**Figure 1:**
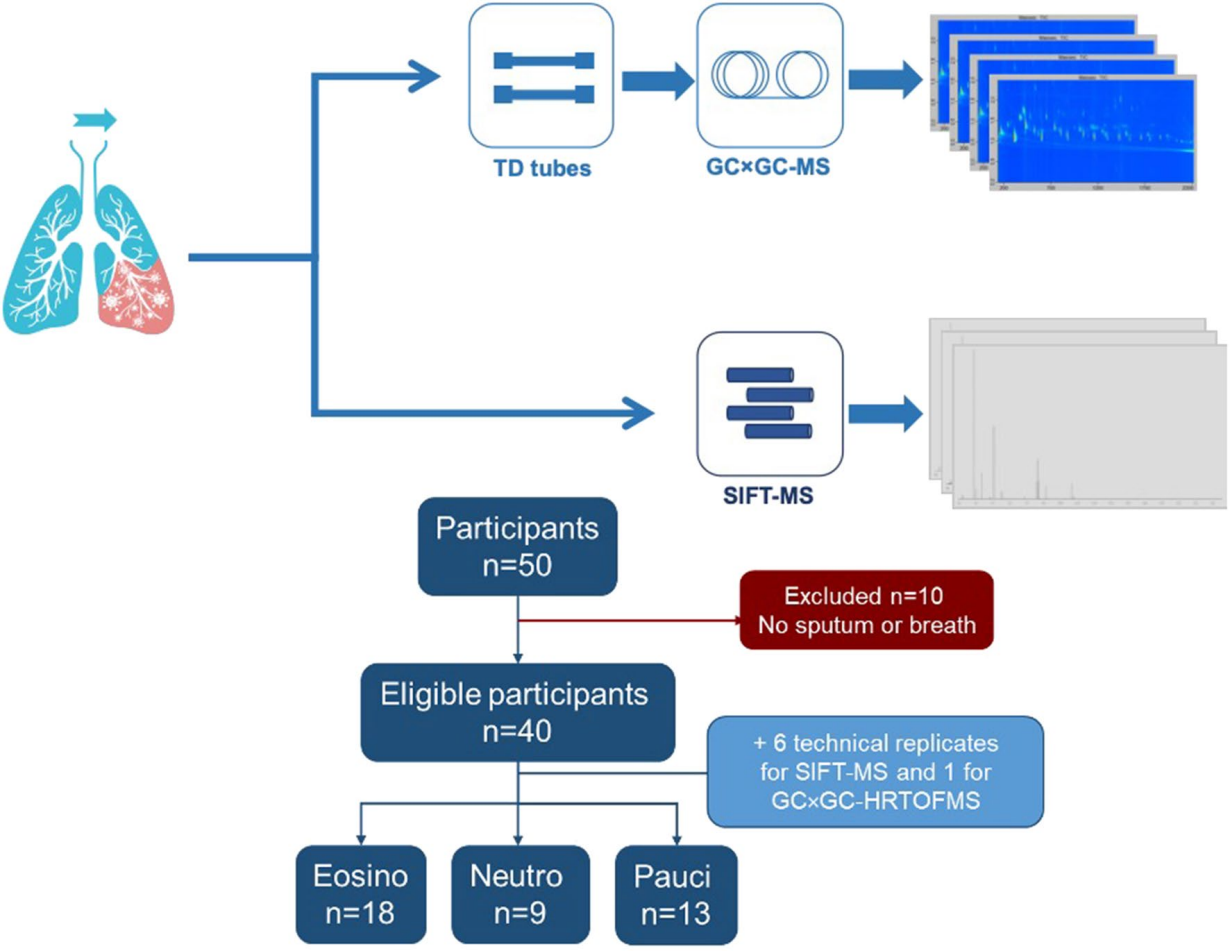
Study analytical design and patient population. Breath samples were collected using gas sampling bags. For GC × GC-HRTOFMS analyses, the bags were transferred onto thermal desorption tubes prior to injection. For SIFT-MS, the bags were directly deflated into the instrument. Data were analyzed using identical processing workflows and outcomes of GC × GC-HRTOFMS and SIFT-MS analyses were judged against each other to serve as a reference in the field. We observed that both approaches offered similar classification capacities.

The aim of this study was to evaluate the complementarity of using untargeted GC × GC-HRTOFMS and SIFT-MS, two commonly used approaches in the field, for exhaled breath analysis. This evaluation was based on the instruments set up and performances to answer a clinical question. To achieve this complementarity evaluation exhaled breath from 50 asthmatic patients were analyzed in parallel by both techniques, with the aim to classify patients according to their asthma phenotype. As a reference, asthma phenotypes were established using sputum analysis. The study design is displayed in Fig. 1.

## Results

### Population characteristics

The patient population characteristics are described in Table 1. This population represents a sub-group of a larger exhaled breath asthma study conducted by Schleich et al.^7^. The studied population contained three phenotypes, namely: eosinophilic, neutrophilic and paucigranulocytic asthmatic patients. The phenotypes were established using the gold standard method, sputum cell count (see method,^7^). In this study, we evaluated the capabilities of GC × GC-HRTOFMS and SIFT-MS to identify eosinophilic patients in an asthma population. The population was balanced between those two groups: eosinophilic (n = 18) vs. other phenotypes (n = 22) (neutrophilic and paucigranulocytic). In Table 1, it can be observed that four parameters were significantly different (*i.e.*, p < 0.05) between the groups. These parameters were fractional exhaled nitric oxide (FeNO) level, blood eosinophils cells (%), sputum eosinophils cells (%), and sputum neutrophils cells (%). These observations were in accordance with the typical asthma population demography. Indeed, FeNO and blood cell count are two complementary approaches to sputum cells count for asthma phenotyping.

**Table 1:** Demographic information for the study population (mean and standard deviation); FEV1 and FeNO (media and min–max range). The p-values for all the demographic information were calculated using chi-squared (for categorical variables) and Wilcoxon–Mann–Whitney rank sum test (for continuous variables). The significant threshold was set at p < 0.05.

Characteristics	Eosinophilic	Others	p-value
N	18	22	
Age	59 (12)	53 (15)	0.153
Gender, F (%)	72	55	0.386
Non-smoker, Y (%)	39	55	0.324
Ex-smoker, Y (%)	44	23	0.145
Smoker, Y (%)	17	23	0.634
Size (cm)	165 (10)	167 (10)	0.355
Weight (kg)	76 (17)	75 (17)	0.744
Atopy (%)	33	41	0.908
ACQ	2 (1)	2 (1.2)	0.0890
ICS therapy, Y (%)	50	32	0.385
FEV1 (% pred)	89.5 (19–123)	87 (58–142)	0.775
FEV1/FVC, %	91 (15.2)	96 (13.3)	0.314
Blood neutrophils, %	54 (5.7)	59 (17.3)	0.193
Blood eosinophils, %	5 (2.1)	2 (2.1)	**3.73E**^**-03**^
FeNO (ppb)	35.5 (6–128)	15 (5–58)	**0.0147**
Sputum neutrophils, %	45 (14.4)	62 (21.2)	**5.35E**^**-03**^
Sputum eosinophils, %	19 (14.1)	1 (1.0)	**7.01E**^**-08**^

ACQ, asthma control questionnaire, ICS therapy, inhaled corticosteroid, FEV1/FVC, Forced expiratory volume in 1 s/Forced vital capacity (FVC).

In addition to this sample set, technical replicates were also generated to evaluate and control the analytical variability. One technical replicate was used for GC × GC-HRTOFMS and six for SIFT-MS. The difference in number is linked to the technical difficulties to obtain replicate samples from patients. For GC × GC-HRTOFMS, the entire Tedlar bag had to be emptied onto the tubes^7^, although for SIFT-MS several consecutive measurements could be carried out from one single bag.

### Unsupervised screening

**Figure 2:**
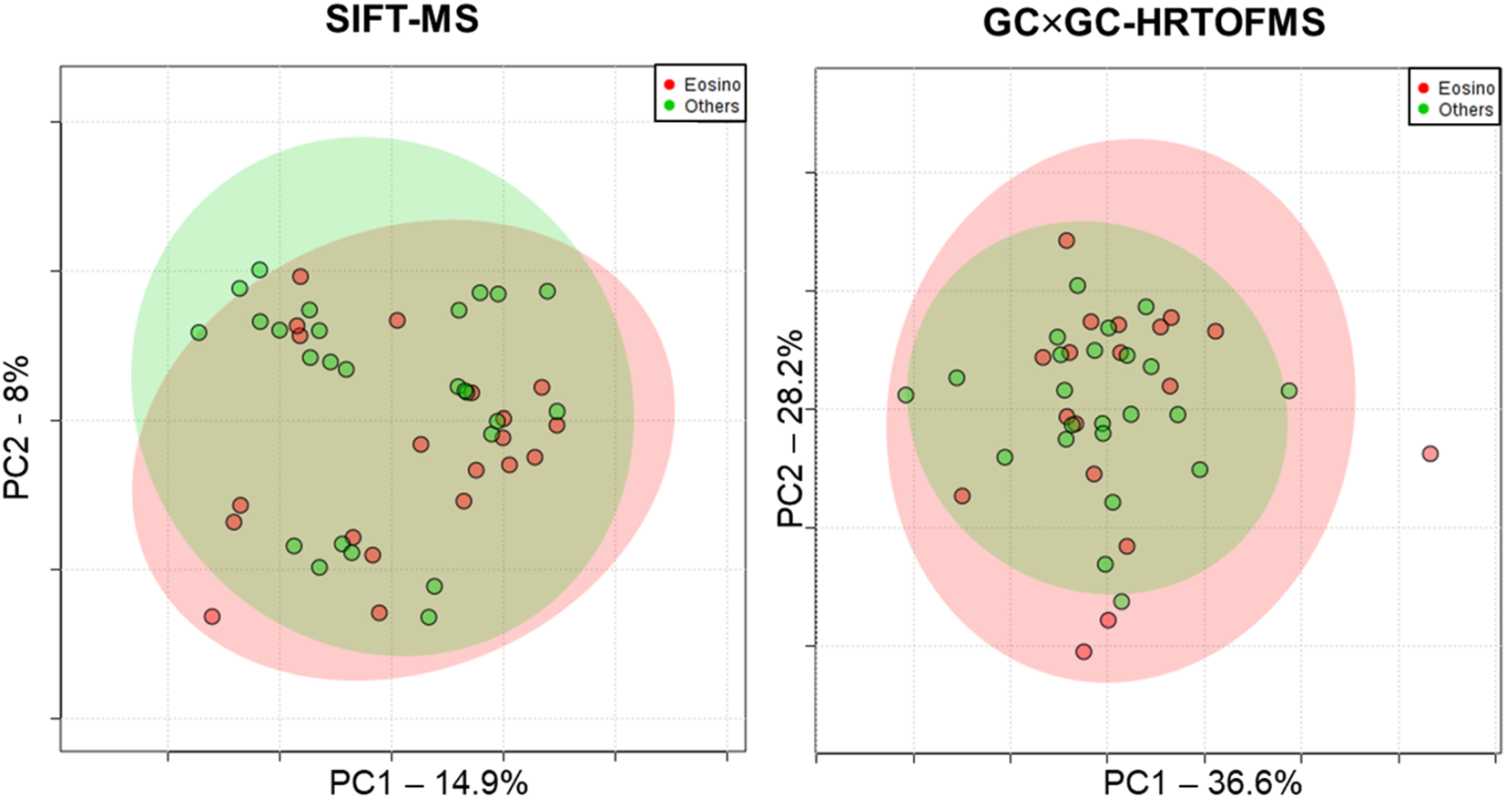
Unsupervised PCA plot for SIFT-MS (left) and GC × GC-HRTOFMS (right) illustrating the absence of outliers or particular clustering trends.

The first step of the pre-processing focuses on the curing of the high-dimensional data sets. For the 40 samples considered, the cleaned raw data matrices contained 456 and 705 features for GC × GC-HRTOFMS and for SIFT-MS, respectively. As these types of data sets are highly susceptible to over-fitting, a careful processing had to be implemented. PCA were performed on both data set to verify the structure of the data and assess the effect of the pre-processing on data integrity (Fig. 2). No particular clustering (e.g. batch effect, population differences) were observed. From this point, data sanity checks were performed. For GC × GC-HRTOFMS, chromatographic artefacts (column bleed, ghost peaks) and related non-representative MS signals, as well as irrelevant known contaminants were removed from data matrices. This allowed to reduce the number of features from 456 to 202. For SIFT-MS, data were cured to remove empty ion channels resulting from the absence of MS signals at certain mass-to-charge (m/z) ratios. This reduced the number of SIFT-MS features to 600. After these data reduction steps, additional PCA visualizations were carried out to validate the procedure (Fig. 2 and Fig. SI-1). The GC × GC-HRTOFMS pre-processing was optimized elsewhere and briefly consisted in probabilistic quotient normalization (PQN), log transform, and auto-scaling^7^. The SIFT-MS data were produced using an untargeted full scan mode on three different precursor ions (H_3_O^+^, O_2_^+^, NO^+^). Full scan SIFT-MS in breath is generally used to reconstruct target information using ion intensity and ion ratio^12^. In this study, the idea is to process every ion channel from each precursor as an independent variable. This approach has been used previously but a clear data processing workflow still needs to be established^13–15^. Such an original approach necessitated the establishment of a dedicated pre-processing strategy. Each ion channel of the raw data was normalized using a unique algorithm (see method). This algorithm ensured the production of a linear and quantitative response from the mass spectrometer for each ion channel, allowing the processing of the full scan matrix without further need for normalization of ion to ion response. Following this step, all empty ion channels were removed from the matrix. Small value imputation was then used for the remaining missing values. Next, signals across all exhaled breath samples were normalized using PQN. This median-based normalization minimized the variability related to the entire sampling procedure. Finally, the data were log-transformed and auto-scaled. As displayed in Fig. 2, the non-supervised structure verification PCA of all the features did not show any apparent clustering. Moreover, the PCA did neither display any potential outlier compounds, ensuring the quality of the pre-processing.

Following the pre-processing optimization, classification accuracy and model, figures of merits were evaluated. In order to compare the results with the previous findings^7^, Random Forest was used as classification algorithm. Based on the study population, we have evaluated the capacity of both analytical methods to identify the eosinophilic phenotype in an asthmatic cohort.

In a first approach, the classification capabilities of Random Forest with no feature selection was evaluated for GC × GC-HRTOFMS and SIFT-MS data set (Table 2). The resulting out of bag (OOB) error was 46.3% and 34.8% for GC × GC-HRTOFMS and SIFT-MS respectively. Moreover, the cross-validation (i.e.*,* permutation test) indicated overfitting of the data due to the high dimensionality. In order to move forward and establish a suitable classification model for each technique, we selected significant features based on their mean decrease accuracy (MDA). All features above 0.0015 MDA were kept for subsequent model building, which resulted in the selection of 10 features and 9 ion channels from the GC × GC-HRTOFMS and SIFT-MS data sets, respectively. For the GC × GC-HRTOFMS features, 6 out of 10 were overexpressed in eosinophilic patients. For SIFT-MS, 7 over 9 ion channels were overexpressed in eosinophilic patients, even if none was significantly different between the two groups using Fisher Ratio or T-test univariate analysis. The intensity pattern can be observed using heat map visualization (Figs. SI-3, SI-4).

**Table 2:** Figures of merit for the different models (unsupervised and supervised) using GC × GC-HRTOFMS and SIFT-MS.

	Unsupervised random forest		Supervised random forest	
	GC × GC-HRTOFMS	SIFT-MS	GC × GC-HRTOFMS	SIFT-MS
Accuracy (%)	53.66	65.22	75.61	76.09
Sensitivity	0.39	0.40	0.72	0.75
Specificity	0.65	0.85	0.78	0.77
Positive predictive value	0.47	0.67	0.72	0.71
Negative predictive value	0.58	0.65	0.78	0.80

### Supervised screening

Based on the selected features from the Random Forest screening, a new classification model was built for each instrumental approach. In order to achieve a robust classification, data were split between training and test sets in a 2:1 ratio. This split was performed to avoid overfitting of the models. Moreover, permutation tests were conducted to ensure the validity of the approach.

**Figure 3:**
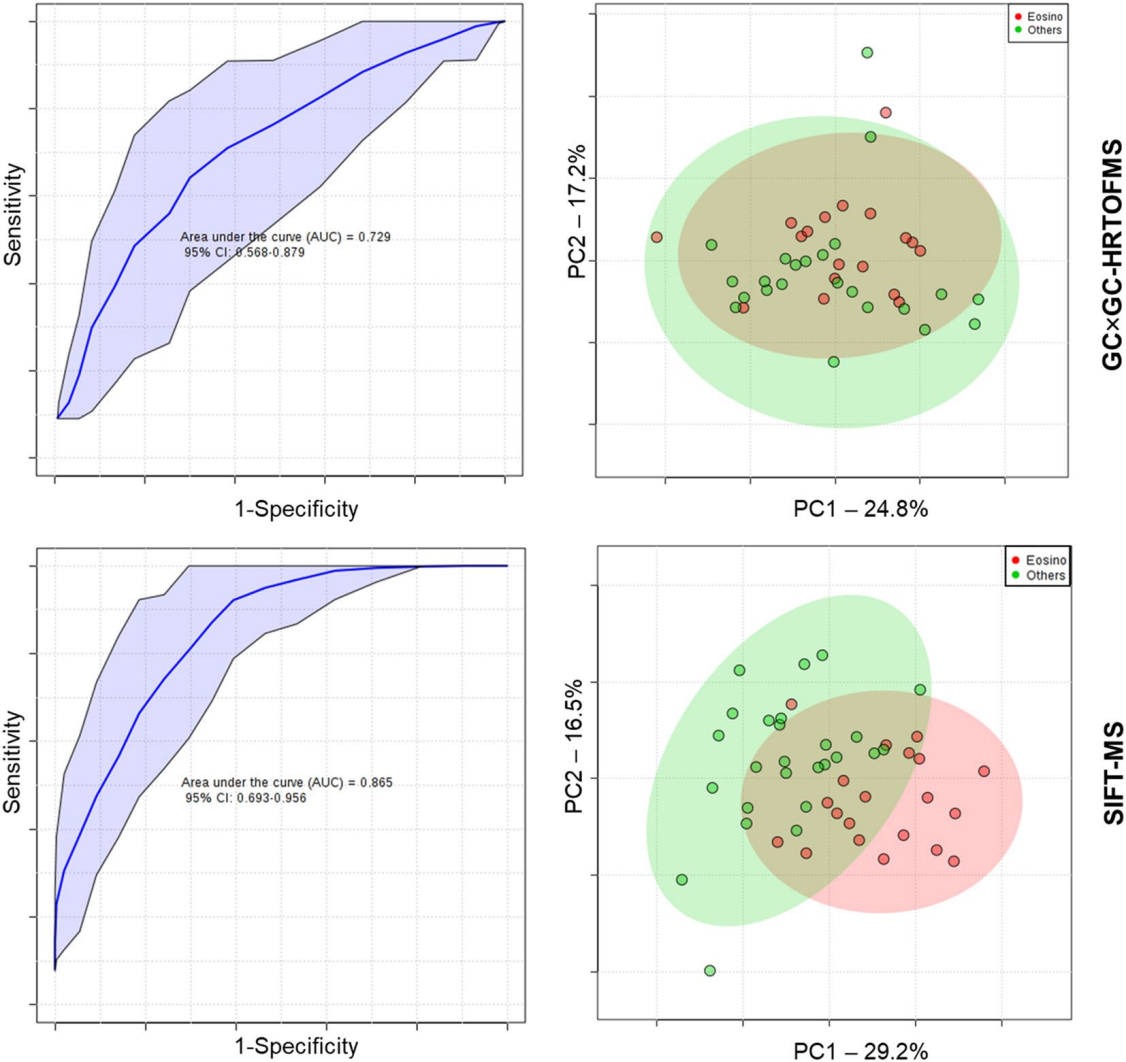
Supervised classification model outcomes using the most significant features (above 0.0015 MDA) using Random Forest algorithm (Top: GC × GC-HRTOFMS; Bottom SIFT-MS). Receiver operating characteristic curves (left) show AUROC values of 73% and 87% for GC × GC-HRTOFMS and SIFT-MS, respectively. PCA score plots (right) depict the apparent differentiation between the eosinophilic phenotype and the others.

For the GC × GC-HRTOFMS classification, 10 significant compounds were used to build the model. The model performances are displayed in Fig. 3. The area under the receiver operating characteristic curve (AUROC) was 73%. In terms of accuracy, the model reached 76%. The other figures of merit are given in Table 2. Based on those selected ion channels, a PCA was built to visualize the separation between the two groups. Compared to the unsupervised PCA (Fig. 2, previous section), a trend between the two groups can be observed when visualizing 42% of the variance. Nevertheless, a strong overlap exists between the two 95% confidence interval ellipses.

For SIFT-MS, 9 significant ion channels were used to build the model (Table 2). The AUROC (87%) was somewhat higher than for GC × GC-HRTOFMS and the model reached 76% of accuracy. Based on those selected ion channels, a PCA was built to visualize the separation between the two groups. Compared to the unsupervised PCA (Fig. 2 and Fig. 3, previous section), the PCA built on the 9 ion channels (Fig. 3) shown a clear trend between the two groups when visualizing 45.7% of the variance.

### GC × GC-HRTOFMS for identification

The 10 selected compounds from the GC × GC-HRTOFMS data set were identified based on different criteria: mass spectra database (match factor and probability), linear retention indices, and accurate mass. An MSI identification confidence Level of 2 or 3 was obtained for all compounds (Table 3)^16^. To reach Level 2, in addition to a mass accuracy below 5 ppm, a compound had to show a mass spectral similarity value above 800 and a probability over 50%. Moreover, in the particular case of nonanal, a pure analytical standard was injected using the exact same GC × GC-HRTOFMS conditions to confirm its identity with an MSI Level 1 confidence. Nonanal was previously identified as an effective biomarker for asthma phenotyping^7^.

**Table 3:** List of the top 10 most significant features (compounds) identified by mean decreased accuracy applying Random Forest algorithm on the GC × GC-HRTOFMS data set. For each feature, important identification metrics are provided.

Feature identification	CAS#	^1^t_R_ (w)	^2^t_R_ (s)	MS library match	MS library probability	MSI level
Butadioic acid dimethyl ester	106-65-0	1410	0.895	917	96.8	2
Nonanal	124-19-6	1542	2.295	930	75.4	1
6-Octen-1-ol, 3,7-dimethyl-, acetate	150-84-5	1982	2.403	942	31.7	3
Cyclopentane, 1,1,3,3-tetramethyl-	50,876-33-0	1210	1.524	760	9.5	3
Decane, 2,5,9-trimethyl	62,108-22-9	990	1.385	730	9.2	3
1,7-Octanediol, 3,7-dimethyl-	107-74-4	1478	2.815	855	17.3	3
5,9-Undecadien-2-one, 6,10-dimethyl-	689-67-8	2178	2.81	930	48.7	3
Acetic acid, phenyl ester	122-79-2	1478	0.93	831	59	2
Diphenyl ether	101-84-8	2106	0.655	823	56.6	2
1-Nonene	124-11-8	982	1.49	925	24.3	3

### SIFT-MS for each precursor

In SIFT-MS, the chemistry taking place behind the ionization process is different for each precursor ion^9^. For this reason, as we worked in untargeted mode, the potential of each precursor ion (in this study: H_3_O^+^, NO^+^, and O_2_^+^) was studied separately and the correlation between them was evaluated. By selecting the significant ion channels for each individual precursor data, and combining them in a single classification model, it was possible to achieve good classification accuracies (Fig. SI-2). Interestingly, there was a complete overlap between 8 of the 9 significant features that were also identified using single precursor processing (see Tables SI-1, SI-2. The potential behind the individual precursor processing and data fusion is of great interest and will be pursued in the future of full scan SIFT-MS development.

## Discussion

First, authors want to state that the present study aimed to evaluate the potential of GC × GC-HRTOFMS and SIFT-MS for breath analysis and exploit their complementarity to advance the exhaled breath research field. Instrument ranking or benchmarking was out of the scope of the study.

GC × GC-HRTOFMS is ideal for untargeted screening of small molecules^8^. It has shown to be a valuable tool in volatolomics^8^ as it can separate and identify (with MSI Level 2 confidence^17^) hundreds of compounds in a single run. It aims to generate chemically resolved data sets to contribute to systems biology of diseases and may provide insights in important metabolic pathways^8^. GC × GC-HRTOFMS comes with a certain level of complexity, which requires a minimum level of expertise to ensure the successful usage of the data sets^8,18,19^. This complexity and the cost make it not directly adapted for daily routine hospital screening but rather for clinical research.

SIFT-MS is a direct-introduction (direct infusion of a gas sample without any chromatographic separation prior to ionization) instrument that rapidly (about 10 times faster than GC × GC-HRTOFMS, i.e. 5 min) provides a pattern-based sample composition. The SIFT-MS used in this study performed soft ionization using three precursor ions (H_3_O^+^, NO^+^, and O_2_^+^) and had the capacity to provide selective mass spectra identification and direct quantification for any compounds of which reaction schemes and associated reaction rate constants are available^10,20^. For this reason, it is an instrument of choice for targeted screening of VOCs^10^. Recently, SIFT-MS has been used for untargeted screening, using full scan MS mode^9,13–15^. It is this untargeted full scan capacity that makes SIFT-MS a potential candidate for pattern-based breath profiling. The instrumental compacity and simplicity of use make SIFT-MS a good candidate as point of care breath screening system.

The complementarity between these two techniques for exhaled breath screening was assessed using a sub-group from a breath-based asthma clinical study^7^.

To compare the classification abilities of both techniques, models were build based on Random Forest algorithms. Random Forest is a machine learning tool based on group of classification trees. This method has demonstrated high stability for high dimensionality data^21^. For both analytical techniques, the models were overfitted, providing poor accuracy. An interesting observation is the high false negative rate (around 0.60) from both models. A potential explanation is the low average probability of classification (0.4–0.6) obtained on the full data matrix. Such a low probability is linked to the high-dimensional structure of our data sets. Indeed, both methods generate hundreds of variables from which most of them are noise. To overpass this limitation, these untargeted models were used to select the most significant features. Moreover, new Random Forest models were built on a training set. Their efficiency and robustness were evaluated on a test set. The data splitting represents a first step in the validation process of the model prior to a replication study. The AUROC value of 73% obtained for GC × GC-HRTOFMS is in agreement with previous studies focusing on the identification of eosinophilic asthma based on the measurement of VOCs in exhaled air^7,22^. For the SIFT-MS, the model generated an AUROC of 87%, which outperformed the GC × GC-HRTOFMS. This performance underlines the high potential for rapid pattern-based screening of asthma population. Nevertheless, all the other figures of merit were similar (Table 2). The lack of large-scale untargeted study on breath using these two techniques makes difficult to compare those numbers with the literature. Indeed, GC × GC-HRTOFMS is relatively new in the field of breath analysis. The AUROC values obtained here are in accordance with performances of other asthma phenotyping methods and further demonstrate the interest of GC × GC-HRTOFMS and SIFT-MS in that context^7,22^.

One of the main advantages of GC × GC-HRTOFMS is its capacity for compound identification. This allows metabolic profiling and has the potential to be a complement in both diagnosis and understanding of the molecular mechanisms involved in the disease occurrence. From the 10 significant features, the identification of nonanal was validated through a neat standard injection. three compounds had a high confidence level of identification (level 2) and the last six had a lower confidence (level 3). These five compounds were identified as highly branched chemicals. This type of molecule usually undergoes strong fragmentation during the electron ionization (EI) process and generates non-specific mass spectra. This is a general problem for the research of biomarkers in breath, where a large number of high potential compounds are often branched^23–25^. The implementation of softer ionization techniques, compared to EI, would reduce fragmentation and preserve higher signals of parent ions, hence improving identification procedures^26,27^. Among the compounds with a high level of identification, different chemical families are present, namely 2 esters, 1 ketone, 1 ether, and 1 aldehyde. Esters and aldehydes have earlier been reported to be possibly generated during lipid degradation in the lung^24^. However, no study has yet clearly identified specific degradation pathways in the context of the production of volatile metabolites. In this list, nonanal has already demonstrated its importance in a previous study^7^. Moreover, it has the second highest MDA from the random forest study. This compound is of high interest as it has been detected in a previous asthma breath study^7^ where it was overexpressed in the non-eosinophilic asthma phenotype. Ongoing studies are investigating the role and origin of nonanal in inflammatory mechanisms.

The SIFT-MS instrument relies on a different technological approach. On the sampling side, SIFT-MS allows direct sample infusion into the MS, which deletes the need of transferring the breath onto a TD tube. TD tubes are highly efficient, but they induce a selectivity bias by trapping compounds in a certain range of volatility. On the technological side, SIFT-MS relies on the utilization to up to 10 chemical precursors to ionize the molecules present in the samples. The positive and negative chemical ionization mechanisms generate soft fragmentation, which maintains identification information. In addition, the combination of different ionization chemistries generates specific fragmentation that make SIFT-MS a powerful tool for targeted analysis^10^. This specificity has also a big added value for untargeted pattern based method, allowing classification model development based on specific ion channels acquired with specific precursors^9^. Due to the technology progress on the precursor’s chemistry and the development of untargeted SIFT-MS analysis, there is a growing need for optimization and processing methods allowing the selection of the best precursors and informative ion channels. Indeed, untargeted full scan SIFT-MS has been used on breath in the past, but the demonstration of its potential and the establishment of the processing workflow still need development. Previous studies used full scan SIFT-MS for correlation calculation with blood markers or to reconstruct target signal, each approach is valid but requires a specific processing workflow^12,13^.

In this study, 9 ion channels were selected to build a classification model. These ions are not significantly different alone but they have a group-based separation capacity (see Figs. SI-3, SI-4). Interestingly, 5 features out of 9 are coming from NO^+^ ionization (see Table SI-1). One could hypothesize that the ionization chemistry of NO^+^ is suitable for the compounds contained in exhaled breath from eosinophilic patients. In addition, the features selected from the SIFT-MS are almost the same regardless the approach used for the selection, namely: individual precursor or full matrix processing (8 over 9 are found, see Tables SI-1, SI-2). This underlines the robustness of this selection process for untargeted SIFT-MS data.

Finally, one could ask why the makers identified by GC × GC-HRTOFMS were not extracted from the SIFT-MS full scan data in order to evaluate their classification capacity through another analytical technique. This direct transfer is challenging because the two techniques rely on different technology (e.g. sample introduction, ionization techniques). It would require model building on exact quantification with both instruments.

This study suffers from two limitations that were accounted for during the analytical workflow in order to minimize their impact on the findings. First, concerning breath sampling, the dual bag approach could have generated some temporal trend in the chemical composition of samples. However, 1st and 2nd bags were randomized prior to GC × GC-HRTOFMS or SIFT-MS analyses. Second, the population size was reasonable but somewhat limited (*i.e.* 50 patients, 40 eligible). Because of this limited number, the feature selection was performed on the same population than the model building. However, compared to other breath studies where sample numbers often range between 20 and 30 patients per group, a set of 40 duplicated patients is reasonable for such a proof-of-concept study^11,28^. In addition, multiple cross-validation and data splitting (between training and test sets) were performed during the data processing to enhance general robustness.

In conclusion, we have demonstrated the efficiency and the complementarity of GC × GC-HRTOFMS and SIFT-MS for asthma phenotyping based on exhaled breath analysis, both techniques provided good classification capacity with their own advantages. On one side, we have a complex chromatographic technique providing a clear view of the sample chemical composition that allows a better understanding of the ongoing metabolic mechanisms. On the other side, SIFT-MS is a rapid, pattern-based screening technique, which has the potential to fulfill the role of clinical tool for breath-based diagnosis.

## Method

### Patient information

The patients included in this study were part of larger clinical validation of breath biomarkers. In short, patients were recruited from the asthma clinic of Liège between October 2014 and April 2018. The recruitment aimed to collect breath sample from 50 patients from which asthma phenotype could be established using sputum granulocytic cell count^29^. The final study population can be found in Table 1. The patient recruitment was based on the STARD guidelines^30^. All the medical information can be found in a previous report^7^.

The study was approved by the Ethics Committee of CHU Liège B70720096732, reference Liège 2009/161 and each patient signed an informed consent. All methods were carried out in accordance with relevant guidelines and regulations.

### Breath sampling

For both analytical approaches, exhaled breath samples were collected using 5 L inert Tedlar bags. Bags were washed twice with high-purity nitrogen (Air Liquide, Liège, Belgium) prior to the sampling, in order to eliminate most of the background. The exhaled breath sampling was performed in the same room across the entire study and background controls were taken. Subjects were asked to inhale, hold their breath for 5 s and subsequently fully exhale into the sampling bag.

Two bags were collected, one for each technique. SIFT-MS samples were injected by connecting the bag directly to the inlet of the instrument. For the GC × GC-HRTOFMS, the content of the bag was transferred onto a thermal desorption (TD) tube prior to be injected into the instrument. The use of a TD tube is required in order to store the sample before injection. Moreover, it provides a pre-concentration of the sample and the possibility to quickly inject into the GC system Fig. 1. The sampling procedure was described by Schleich and colleagues^7^.

### Analytical instrumentation and parameters

#### GC × GC-HRTOFMS analytical conditions

All samples were thermally desorbed using a MARKES TD 100 XR (MARKES, UK). TD tubes (Tenax TA-Carbopack B) were heated at 290 °C for 5 min with a split of 20:1. The split fraction was recollected on the tube for potential second analysis. The chromatographic separation was performed on a Pegasus GC-HRT 4D (LECO Corporation, St. Joseph, MI, USA) equipped with a secondary oven and a quad-jet, dual-stage thermal modulator. Acquisition was controlled using ChromaTOF-HRT version 4.2.3.1. The column set was made of two capillary columns connected, a Rxi-624Sil MS (30 m × 0.25 mm i.d., 1.40 µm d_f_) combined with a Stabilwax (Restek Corporation) (2 m × 0.25 mm i.d., 0.50 µm d_f_). The connection between the two columns was made using a SilTite µ-union (Trajan Scientific and Medical, Australia). A constant helium flow of 1.00 mL/min was used throughout the run. The initial main temperature oven was 35 °C and was increased to 240 °C at a rate of 5 °C/min (total 51 min). The secondary oven offset was + 5 °C in relation to the primary oven temperature and the modulator offset was + 15 °C in relation to the secondary oven. A 4 s modulation period (P_M_) was used with a 0.80 s hot pulse time. The MS transfer line was held at 220 °C. An acquisition delay of 300 s was used. The mass acquisition range was 29–450 m*/z* with an acquisition rate of 200 Hz and a 2 kHz extraction frequency. The ion source temperature was 250 °C. The electron ionization energy was 70 eV and an emission current of 1.0 mA was used. The detector was tuned prior to each sequence. The mass resolution achieved during each tune always exceeded 30,000. The tune compound valve was opened during the entire run duration for constant mass calibration using perfluorotributylamine (PFTBA).

A full validation and quality control procedure was performed during the study using seven compounds representing important asthma biomarkers. All control charts and figures of merit are available in a previous publication^7^.

#### SIFT-MS analytical conditions

For the SIFT-MS analysis, the Tedlar bag containing the exhaled breath sample was directly connected to the heated instrument inlet (Voice200 Ultra, Syft Technologies, Christchurch, New Zealand). The inlet is equipped with a capillary restriction allowing controlled introduction of the breath sample into the flow tube by means of the instrument vacuum. The reagent ions (H_3_O^+^, NO^+^ and O_2_^+^) were generated by a microwave air discharge at 0.5 Torr and selected by means of a short quadrupole mass filter after which they are consecutively injected into the flow tube in a stream of Helium as carrier gas. In the flow tube, sample analytes and precursor ions combine giving rise to unique product ions, which are separated from each other by means of a second quadrupole. The instrument was applied in full scan mode using three acquisition repeats in a single run in the mass-to-charge ratio (m/z) range of 15–250 amu and a dwell time of 100 ms. The full scan data (ion counts per second, cps) averaged over the sampling time for each m/z value was used for the statistical analyses.

### Data pre-processing

#### Chromatographic alignment and feature identification

All the raw data were exported in a netCDF format from the ChromaTOF-HRT software. They were process using the GC Image HR 2.5 software suites (Zoex Corporation, Houston, TX, USA). A specific GC Image-based process was then developed using GC Project and Image investigator features. First, a cumulative image was generated, and the peak detection parameters were set, using a minimum area of 150 was performed. The detected features were then tentatively identified based on the mass spectra using the National Institute of Standards and Technology (NIST) 2014 and Wiley 10 libraries with a match factor threshold of minimum 800. Moreover, the identification was reinforced by the accurate mass information. From there, a template was built and all contaminant compounds (e.g. column bleed and background compounds) were removed. This template was then used to align all samples. The resulting peak table was finally exported for further statistical processing in a third-party.

#### SIFT-MS signal normalization

The SIFT-MS output data was normalized using Syft Technologies proprietary algorithm. In brief, each individual ion channel is normalized to a linear quantitative signal that takes into account both reagent ion and product ion yields in function of lens voltages, temperature and molecular weight. More information regarding the normalization can be found in supplementary materials. The capacity of SIFT-MS to generate quantitative results without external calibration represents a huge advantage in the context of clinical implementation. Moreover, it ensures direct comparison of every ion channels during the statistical processing, all sample to sample variations are coming from the sample itself.

### Multivariate analysis

**Figure 4:**
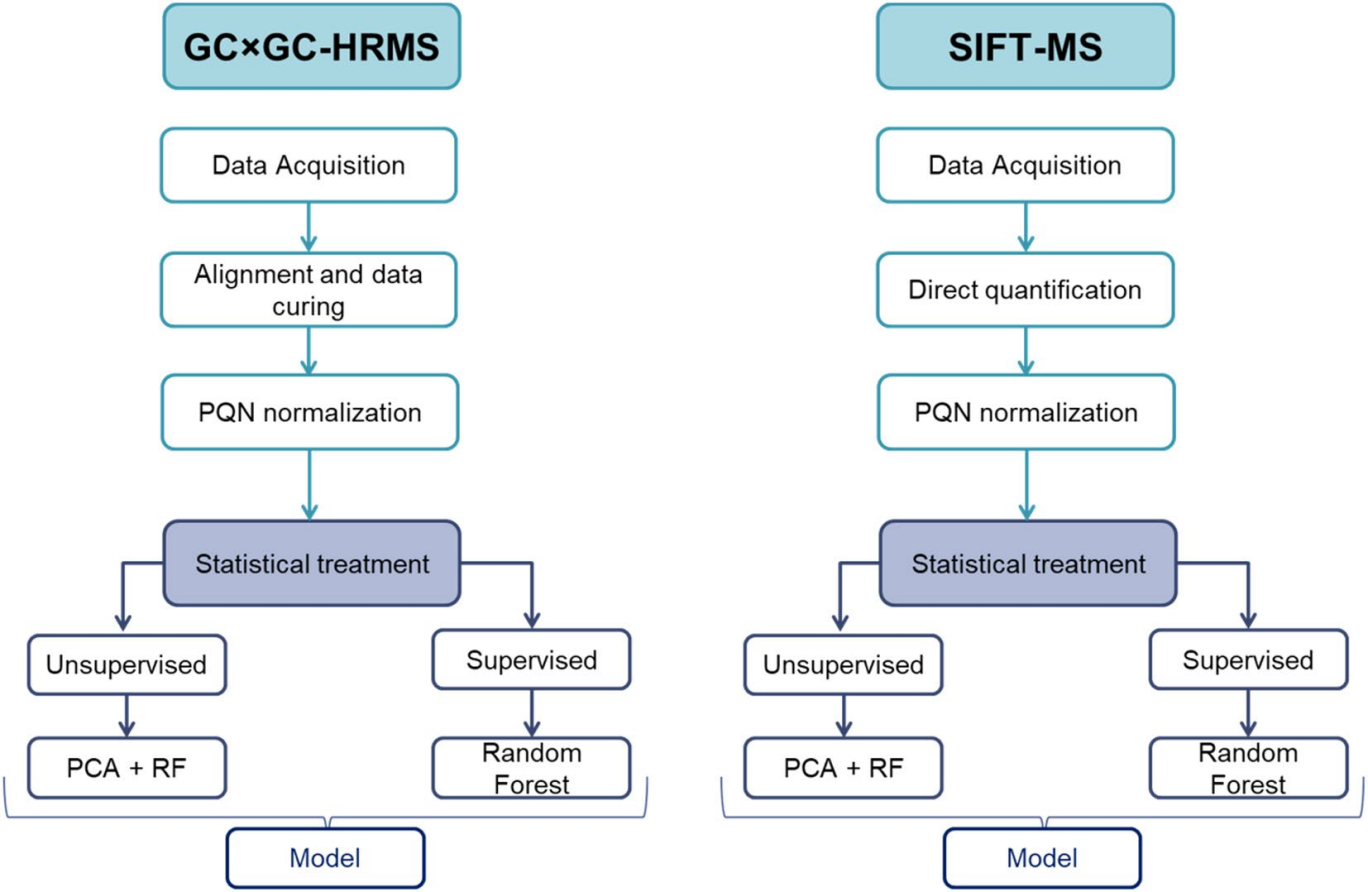
Data processing workflow for SIFT-MS and GC × GC-HRTOFMS. The same design was applied for both techniques, but specific pre-processing was conducted.

Based on the unbalanced patient population, the study was focused on the evaluation of the capability of the two analytical techniques for the discrimination of the eosinophilic phenotype from the rest of the asthmatic population, *i.e.* paucyganulocitic and neutrophilic phenotypes. This rule in approach can be easily compared with other asthma diagnostic approaches, such as FeNO or blood cell count^6,7^. Moreover, to be able to compare the classification capacity of both approaches, a common processing workflow was designed (Fig. 4). The data processing workflow contains two main parts: unsupervised and supervised screenings. The first one aims to evaluate the structure of the data obtained with each analytical technique. The second one evaluates the classification accuracy of the models for each instrumental technique. To achieve that, we used Random Forest classification algorithms for supervised and unsupervised classification. Due to the limited population of the study, different cross validation steps were included. First, the figure of merits from the different models were obtained after splitting the data between a training and a test set. Moreover, random sample labeling, and feature picking were tested. These two tests provided classification metrics corresponding to random assignment (around 50%).

The data processing was conducted using in the open source software for statistics, R (The R Foundation for Statistical Computing, V3.4.3, Vienna, Austria) and the online platform MetaboAnalyst^31^.

## Supplementary information

Supplementary information.

## Data Availability

The data described in the manuscript are available on reasonable request to the corresponding author.
