# Supplementary material for: Multimodal combination of GC × GC-HRTOFMS and SIFT-MS for asthma phenotyping using exhaled breath
Source: Sci Rep. 2020 Sep 30;10:16159. doi: 10.1038/s41598-020-73408-2 (PMC7528084; doi:10.1038/s41598-020-73408-2)
Supplement: Supplementary file 1 — Supplementary information. [file 41598_2020_73408_MOESM1_ESM.docx]

**MULTIMODAL COMBINATION OF GC×GC-HRTOFMS AND SIFT-MS FOR ASTHMA PHENOTYPING**

P.-H. Stefanuto^1*^, D. Zanella^1^, J. Vercammen^2,3^, M. Henket^4^, F. Schleich^4^, R. Louis^4^, J.-F. Focant^1^

^1^ Organic and Biological Analytical Chemistry Group, MolSys research unit, University of Liège, Liège, Belgium

^2^ Interscience, Avenue J.E. Lenoir, Louvain-la-Neuve, Belgium

^3^ Engineering, Industrial Catalysis and Adsorption Technology (INCAT), Ghent University, Ghent, Belgium

^4^ Pneumology and Allergology, GIGA Research Group, CHU of Liège, University of Liege, Liège, Belgium

* Corresponding Author

PH Stefanuto, PhD

University of Liège

Organic & Biological Analytical Chemistry Group, MOLSYS Research Unit

Allée du 6 Août B6c, B-4000 Liège, Belgium

Phone: +32 (0)4 366 36 05

Fax: +32 (0)4 366 43 87

email: ph.stefanuto@uliege.be


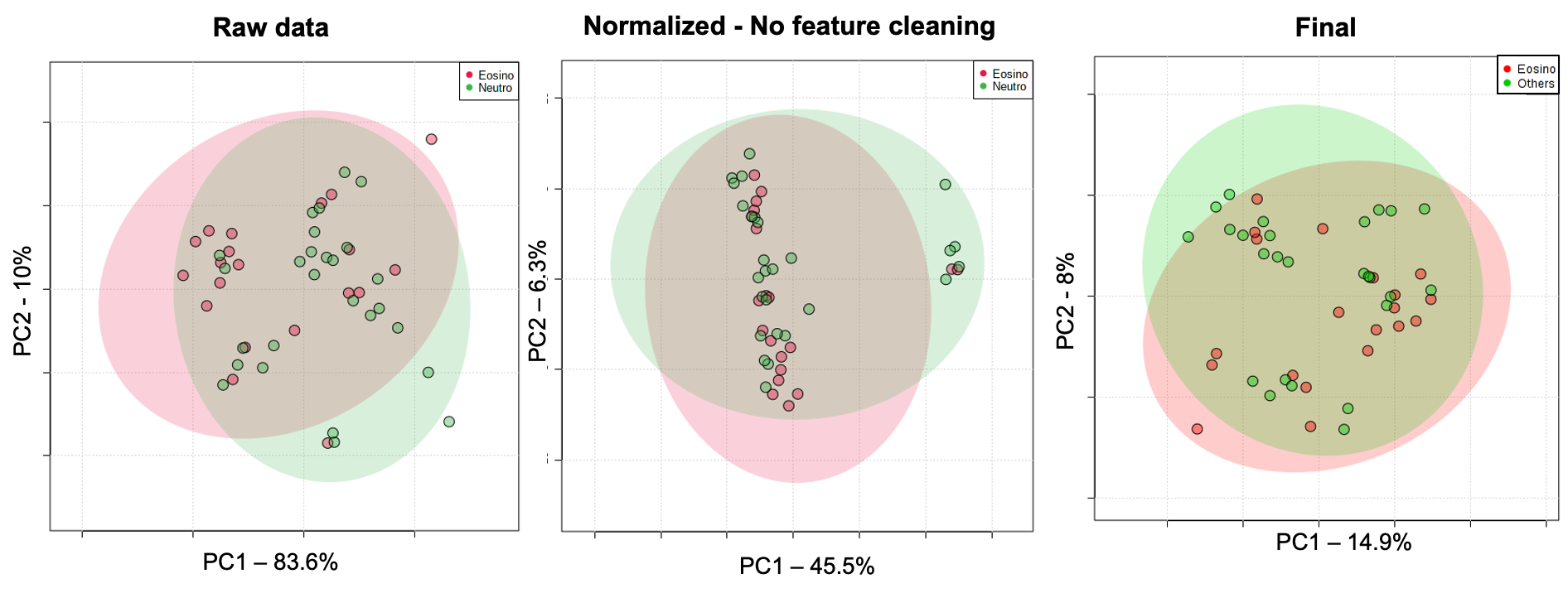


**Figure SI-1:** Unsupervised PCA at different pre-processing step on the SIFT-MS data. The final PCA is the one from Figure 2.


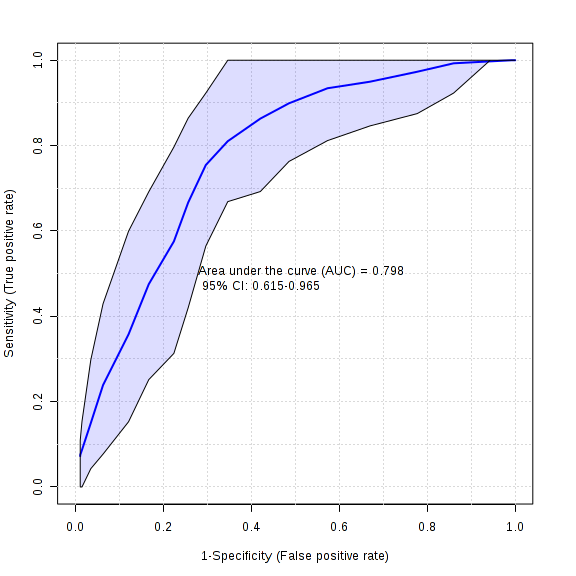


**Figure SI-2:** AUROC from the supervised screening using single precursor processing (see Table SI-2).

**Table SI-1:** Precursors selected during the group processing (all precursors together) using Random Forest.

| **Group processing** | |
| --- | --- |
| **Precursors** | **m/z** |
| H_3_O^+^ | 133 |
| H_3_O^+^ | 208 |
| NO^+^ | 54 |
| NO^+^ | 73 |
| NO^+^ | 97 |
| NO^+^ | 99 |
| NO^+^ | 114 |
| O_2_^+^ | 28 |
| O_2_^+^ | 126 |

**Figure SI-3:** Heat map displaying the signal intensity for the ion channels selected during the global processing (see Table SI-1).

**Table SI-2:** Precursors selected during the single precursor processing using Random Forest.

| **Single precursor processing** | |
| --- | --- |
| **Precursors** | **m/z** |
| H_3_O^+^ | 32 |
| H_3_O^+^ | 34 |
| H_3_O^+^ | 73 |
| H_3_O^+^ | 109 |
| H_3_O^+^ | 114 |
| H_3_O^+^ | 133 |
| H_3_O^+^ | 134 |
| H_3_O^+^ | 139 |
| H_3_O^+^ | 160 |
| H_3_O^+^ | 208 |
| NO^+^ | 50 |
| NO^+^ | 54 |
| NO^+^ | 63 |
| NO^+^ | 66 |
| NO^+^ | 73 |
| NO^+^ | 82 |
| NO^+^ | 83 |
| NO^+^ | 97 |
| NO^+^ | 99 |
| NO^+^ | 163 |
| O_2_^+^ | 28 |
| O_2_^+^ | 32 |
| O_2_^+^ | 70 |
| O_2_^+^ | 71 |
| O_2_^+^ | 77 |
| O_2_^+^ | 85 |
| O_2_^+^ | 104 |
| O_2_^+^ | 126 |

**Figure SI-4:** Heat map displaying the signal intensity for the ion channels selected during the single precursor processing (see Table SI-2).

**SIFT-MS Normalization algorithms**

Usually, SIFT-MS is used in target mode, in which a limited list of components of interest is measured in real time. A high interest of SIFT-MS is that it does not require external calibration since it is able to compensate for factors that have an immediate effect on detection accuracy and reproducibility, such as molecular weight of the formed product ion, lens voltages, vacuum conditions, temperature, etc. Particularly the molecular weight of the product ion is important. Since higher molecular weight travel slower through the quadrupole, the corresponding signal will exhibit a negative bias when not compensated for. This normalization is important since a single component that gives rise to product ions with different molecular weight should result in the same concentration of the native analyte. In order to achieve this goal, SIFT-MS uses mass-dependent instrument calibration factors (ICFs), which are calculated on a daily basis prior to starting an analysis sequence. Therefore, a standard reference mixture is measured that contains a variety of components with known concentrations that span a broad molecular weight range. An ICF is calculated for each component (i.e., product ion mass) in the mixture taking into account its concentration in the standard mixture and the reaction rate constant of the reaction of the component with a particular precursor ion.

More information can be found in Smith, D. & Patrik, S. Selected ion flow tube mass spectrometry (SIFT-MS) for on-line trace gas analysis. Mass Spectrom. Rev. 24, 661–700 (2005).
